# Layer specificity of inputs from supplementary motor area and dorsal premotor cortex to primary motor cortex in macaque monkeys

**DOI:** 10.1038/s41598-019-54220-z

**Published:** 2019-12-03

**Authors:** Taihei Ninomiya, Ken-ichi Inoue, Eiji Hoshi, Masahiko Takada

**Affiliations:** 1 Systems Neuroscience Section, Primate Research Institute, Kyoto University, Inuyama, Aichi 484-8506 Japan; 2 Japan Science and Technology Agency (JST), Core Research for Evolutional Science and Technology (CREST), Tokyo, 102-0076 Japan; 3 Department of Developmental Physiology, National Institute for Physiological Sciences, Okazaki, Aichi 444-8585 Japan; 4 Japan Science and Technology Agency (JST), Precursory Research for Embryonic Science and Technology (PRESTO), Tokyo, 102-0076 Japan; 5 Frontal Lobe Function Project, Tokyo Metropolitan Institute of Medical Science, Setagaya-ku, Tokyo, 156-8506 Japan

**Keywords:** Motor cortex, Neural circuits

## Abstract

The primate frontal lobe processes diverse motor information in parallel through multiple motor-related areas. For example, the supplementary motor area (SMA) is mainly involved in internally-triggered movements, whereas the premotor cortex (PM) is highly responsible for externally-guided movements. The primary motor cortex (M1) deals with both aspects of movements to execute a single motor behavior. To elucidate how the cortical motor system is structured to process a variety of information, the laminar distribution patterns of signals were examined between SMA and M1, or PM and M1 in macaque monkeys by using dual anterograde tract-tracing. Dense terminal labeling was observed in layers 1 and upper 2/3 of M1 after one tracer injection into SMA, another tracer injection into the dorsal division of PM resulted in prominent labeling in the deeper portion of layer 2/3. Weaker labeling was also visible in layer 5 in both cases. On the other hand, inputs from M1 terminated in both the superficial and the deep layers of SMA and PM. The present data indicate that distinct types of motor information are arranged in M1 in a layer-specific fashion to be orchestrated through a microcircuit within M1.

## Introduction

Even a simple motor command is generated through many brain regions. Accumulated evidence to date indicates that the primate motor system operated in the frontal lobe is composed of multiple areas to deal with various aspects of movements in parallel (for reviews, see refs. ^1–6^). For example, the supplementary motor area (SMA) is related to internally-triggered movements, whereas the premotor cortex (PM) is to externally-guided movements. The primary motor cortex (M1) is involved in both aspects of movements and constitutes the major node that sends voluntary motor commands toward the spinal cord. It is well known that the basal ganglia (BG) and the cerebellum (Cb), each of which has strong connectivity with the frontal motor-related areas via the thalamus, also exert critical roles in motor control. From the functional and hodological viewpoints, there is a consensus that BG and Cb, rather relevant to internally-triggered or externally-guided motor behavior, are linked more tightly to SMA or PM, respectively^1,6,7^.

Anatomical and electrophysiological studies have shown that BG and Cb convey signals to different layers of M1 via distinct thalamic nuclei: BG-derived signals are directed mainly toward layer 1, whereas Cb-derived signals are directed toward layer 2/3 as well as toward deeper layers^8–20^. The segregation of signals from BG and Cb toward M1 implies that the integration of motor information takes place in an organized fashion within M1. With respect to the intercortical networks around SMA, PM, and M1, their laminar origins have extensively been investigated in macaque monkeys by using retrograde labeling technique^21–27^. These studies have demonstrated that the neurons giving rise to interconnections among SMA, PM, and M1 are located in both the superficial (1–3) and the deep (5 and 6) layers, leading to a conclusion that there is no bias in the direction of information flow across the three motor-related areas. By contrast, the layer-specific pattern of terminal distribution of each component projection remains to be fully understood, although some anatomical data are available^21,28–30^. Hence, an open question arises as to how the motor-related areas of the frontal lobe are structurally organized to process functionally distinct signals. To address this issue, the intercortical connectivity among SMA, PM (especially its dorsal division; PMd), and M1 was examined in terms of the layer specificity by using dual anterograde tract-tracing.

## Results

### Laminar distribution of SMA-derived terminals in M1

**Figure 1:**
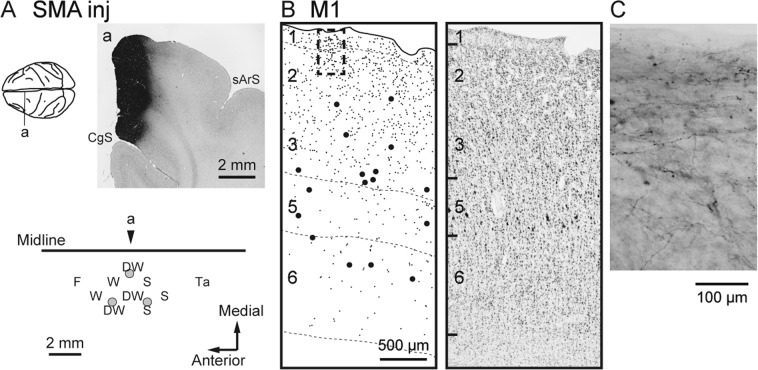
Anterograde and retrograde labeling in M1 after BDA injections into SMA (monkey B). (**A**) Upper, Representative coronal section (**a**) through the injection site (right). CgS, cingulate sulcus; sArS, superior limb of the arcuate sulcus. The approximate anteroposterior level of the section (**a**) is indicated in the dorsal view of the brain (left). Lower, Result of intracortical microstimulation (ICMS) mapping. Filled gray circles denote the locations of the injection sites. In the somatotopic map, the body parts of which movements were evoked by ICMS are indicated as follows: D, digits; F, face; S, shoulder; Ta, tail; W, wrist. The combined letter, DW, represents the locus where different body-part movements were elicited in that order during microelectrode penetration. The arrowhead indicates the approximate anteroposterior level of the section (**a**) in the somatotopic map. (**B**) Representative coronal section showing the laminar organization of anterograde and retrograde labeling in M1 (left). Each small or large dot corresponds to one terminal varicosity or cell body labeled with BDA, respectively. Cortical layers are depicted on the left side of the section and demarcated with broken lines. A corresponding Nissl-stained section is shown on the right. (**C**) Higher-magnification photomicrograph showing BDA-labeled fibers, taken from the rectangular area in B.

**Figure 2:**
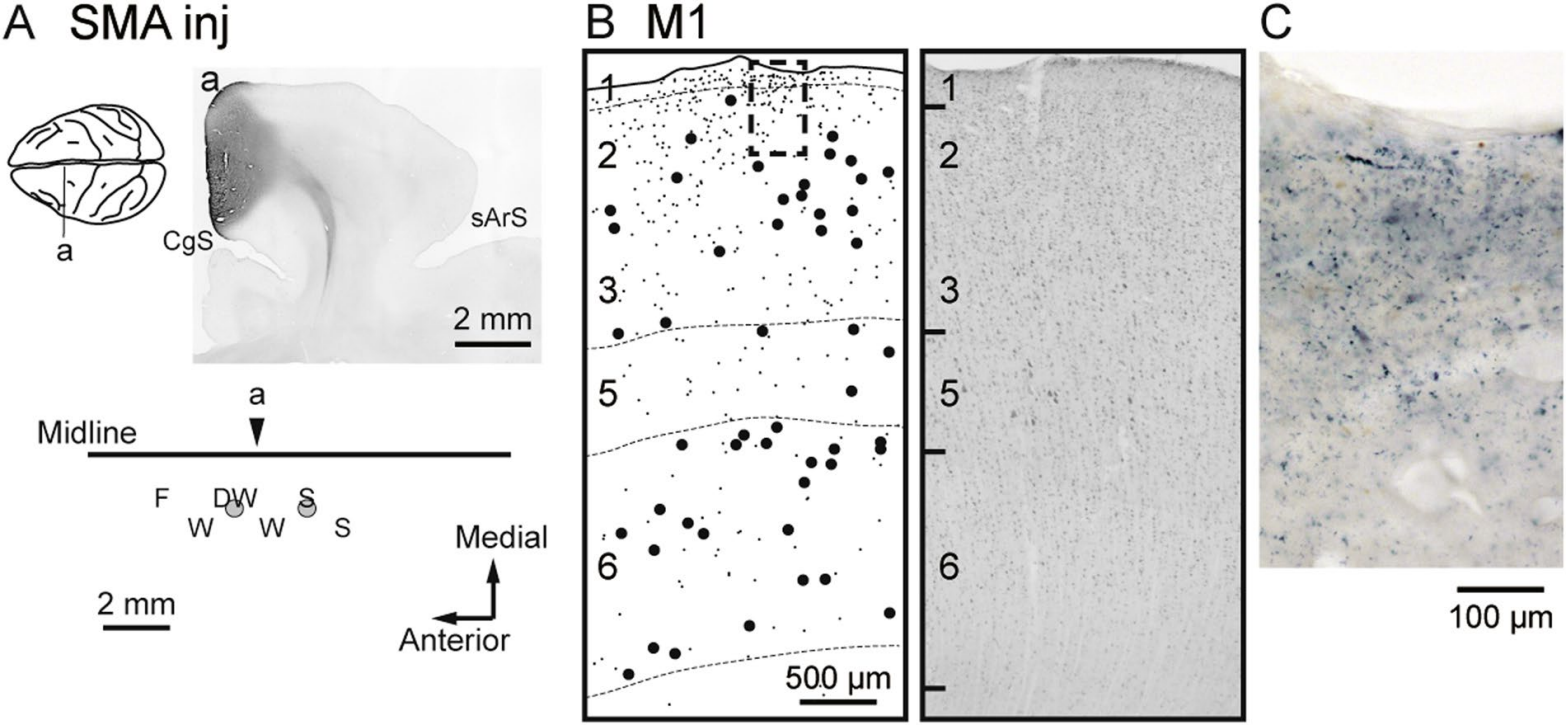
Anterograde and retrograde labeling in M1 after WGA-HRP injections into SMA (monkey M). All conventions and abbreviations are as in Fig. 1.

**Figure 3:**
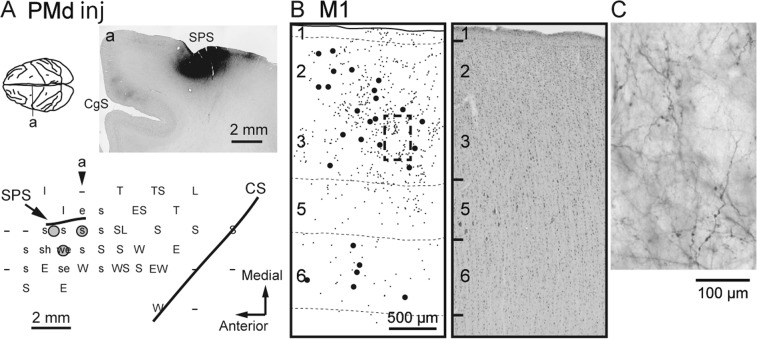
Summary plots of anterograde labeling. Left, Averaged labeling density across M1 layers for the SMA (blue; n = 17) and PMd (red; n = 19) projections. Right, Averaged labeling density across SMA (blue; n = 19) or PMd (red; n = 22) layers for the M1 projection. Arrowheads denote the locations of the laminar borders averaged across the analyzed sites. The lighter shaded colors represent ± 1 SD.

We first assessed the laminar distribution of anterograde labeling in M1 after injecting biotinylated dextran amine (BDA) or wheat germ agglutinin-conjugated horseradish peroxidase (WGA-HRP) into SMA (monkeys B and M, respectively; Table 1). In both cases, the injection sites were placed in the forelimb region of SMA according to the somatotopic map obtained with the intracortical microstimulation (ICMS) (Figs. 1A and 2A). The tracer injections into SMA resulted in dense clusters of labeled terminals in M1, just anterior to the central sulcus where the forelimb was represented^31,32^. While a certain amount of terminal labeling was seen in the caudal part of M1, especially in the anterior bank of the central sulcus (so-called “new” M1^33,34^), the densest labeling was found in the precentral gyrus. Therefore, anterograde labeling in the precentral gyrus was mainly chosen for further analyses. As shown in Fig. 1B,C and 2B,C, prominent labeling was observed in layers 1 and 2/3. Terminal labeling in layer 2/3 was often extended throughout its entire superficial-deep part with the peak density at the middle to superficial level (see also Fig. 3). Weaker labeling was also found in layer 5 where corticospinal neurons were located, and layer 6 was virtually devoid of labeling.

**Table 1:** Locations of the cortical injections and tracers employed in the experiments.

Monkey	Hemisphere	Injection site	Tracer	Amount (μL)
A	Right	M1	BDA	3 × 1.0
B	Left	SMA	BDA	3 × 1.0
	Right	PMd	WGA-HRP	3 × 0.1
G	Left	M1	WGA-HRP	3 × 0.1
H	Left	PMd	BDA	3 × 1.0
M	Left	SMA	WGA-HRP	2 × 0.1

The averaged laminar distribution pattern in M1 of terminal labeling from SMA in monkeys B and M (11 and 9 sites examined, respectively; Table 1) is shown in Fig. 3. Layer 1 and the upper part of layer 2/3 had the heaviest labeling, and, also, the upper part of layer 5 appeared to have some labeling. By contrast, the deeper layers contained only sparse labeling. It should be mentioned here that the input from the pallidum of BG reaches layer 1 of M1 via the thalamus^11,16,17,19,20^, and, therefore, functionally-relevant signals derived from SMA and BG might terminate within the same layer of M1.

As the present study focuses on analyzing anterograde labeling and comprehensive prior works already reported the connectivity of the motor-related areas with the aid of retrograde tracers^21–27^, the patterns of retrograde labeling for monkeys B and M and, also, for the other subjects will be summarized briefly at the end of the Results section and in Table 2.

**Table 2:** Laminar distribution of labeled neurons in each case.

Monkey	Injection site	% superficial		
		M1	SMA	PMd
A	M1	—	59.3 (380/641)	56.0 (772/1379)
B	SMA	57.7 (472/818)	—	—
	PMd	50.5 (1141/2259)	—	—
G	M1	—	55.7 (565/1014)	52.8 (1179/2232)
H	PMd	48.2 (575/1192)	—	—
M	SMA	59.0 (819/1389)	—	—

Numbers in parentheses denote numbers of labels in the superficial layers and total numbers of labels.

### Laminar distribution of PMd-derived terminals in M1

**Figure 4:**
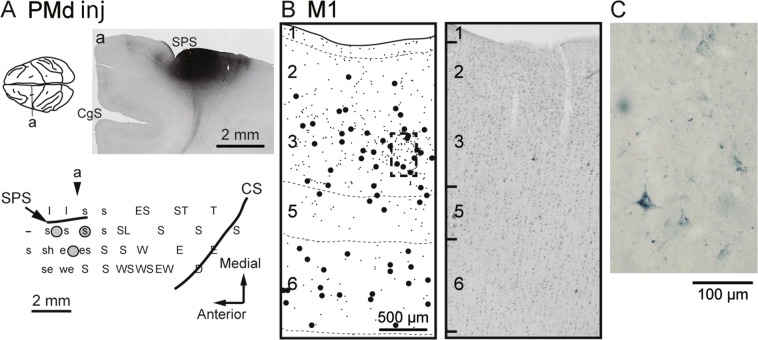
Anterograde and retrograde labeling in M1 after BDA injections into PMd (monkey H). (**A**) Upper, Representative coronal section (a) through the injection site (right). SPS, superior precentral sulcus. Lower, Result of ICMS mapping. In the somatotopic map, the body parts of which movements were evoked by ICMS are indicated as follows: E (e), elbow; L (l), leg; S (s), shoulder; T; trunk; W (w), wrist. The uppercase and lowercase letters represent evoked movements by ICMS with a train of 11 or 44 current pulses, respectively. –, no movement elicited. CS, central sulcus. (**B**) Representative coronal section showing the laminar organization of anterograde and retrograde labeling in M1. A corresponding Nissl-stained section is shown on the right. (**C**) Higher-magnification photomicrograph taken from the rectangular area in B. Other conventions and abbreviations are as in Fig. 1.

**Figure 5:**
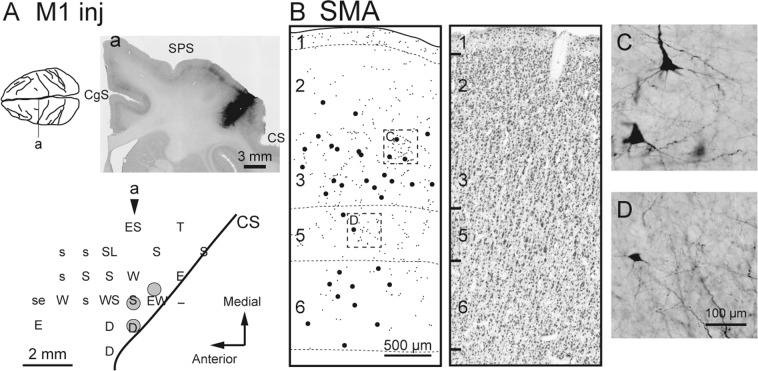
Anterograde and retrograde labeling in M1 after WGA-HRP injections into PMd (monkey B). All conventions and abbreviations are as in Figs. 1 and 4.

The laminar distribution of anterograde labeling in M1 after the tracer injections into PMd was then examined in two monkeys (monkeys B and H; Table 1). The BDA (monkey B) and WGA-HRP (monkey H) injections were made just lateral to the superior precentral sulcus where the shoulder was represented based on the result of ICMS mapping (Figs. 4A and 5A). In each case, dense terminal labeling was observed in the precentral gyrus with moderate labeling in the anterior bank of the central sulcus. Although the somatotopy was not extensively surveyed in M1, the major labeling site corresponded to the shoulder region in the precentral gyrus, thus indicating the existence of a somatotopically-organized projection from PMd to M1. Like the SMA-derived terminals in M1, anterograde labeling in the precentral gyrus was chiefly chosen for further analyses. Figures 4B,C and 5B,C show representative laminar patterns of the PMd-derived terminals in M1. Accumulations of labeled terminals were found mostly in layer 2/3, especially in its deeper part (see also Fig. 3). Terminal labeling in layer 1 was much weaker than in the SMA-injection cases. In addition, there was an explicit labeling in the upper part of layer 5, but layer 6 only contained sparse labeling.

The averaged laminar distribution pattern in M1 of terminal labeling from PMd in monkeys B and H (12 and 10 sites examined, respectively; Table 1) is shown in Fig. 3. As described above, marked labeling was seen in layer 2/3 with the peak density in its deeper part, thus forming a striking contrast to the SMA input to M1. Consistent with the SMA-derived terminals in M1, the upper part of layer 5 also contained weaker labeling. Anterograde labeling from PMd was only sparsely detected in other layers. It should be emphasized here that the deep layer 3 of M1 receives strong cerebello-thalamo-cerebral inputs^8–10,16,20^. Hence, functionally-related signals generated in PMd and Cb appear to reach the same layer of M1.

### Laminar distributions of M1-derived terminals in SMA and PMd

**Figure 6:**
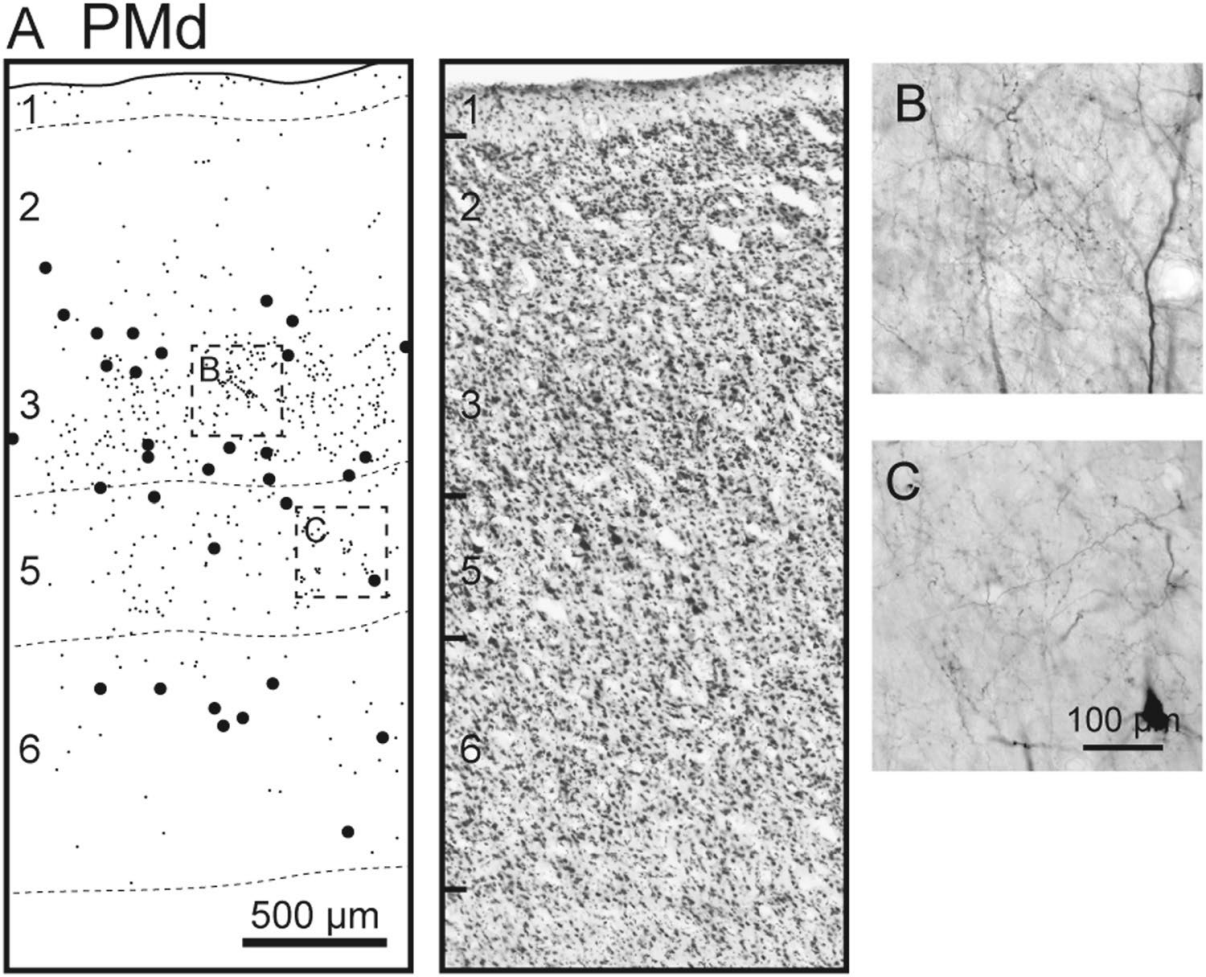
Anterograde and retrograde labeling in SMA after BDA injections into M1 (monkey A). All conventions and abbreviations are as in Figs. 1 and 4.

**Figure 7:**
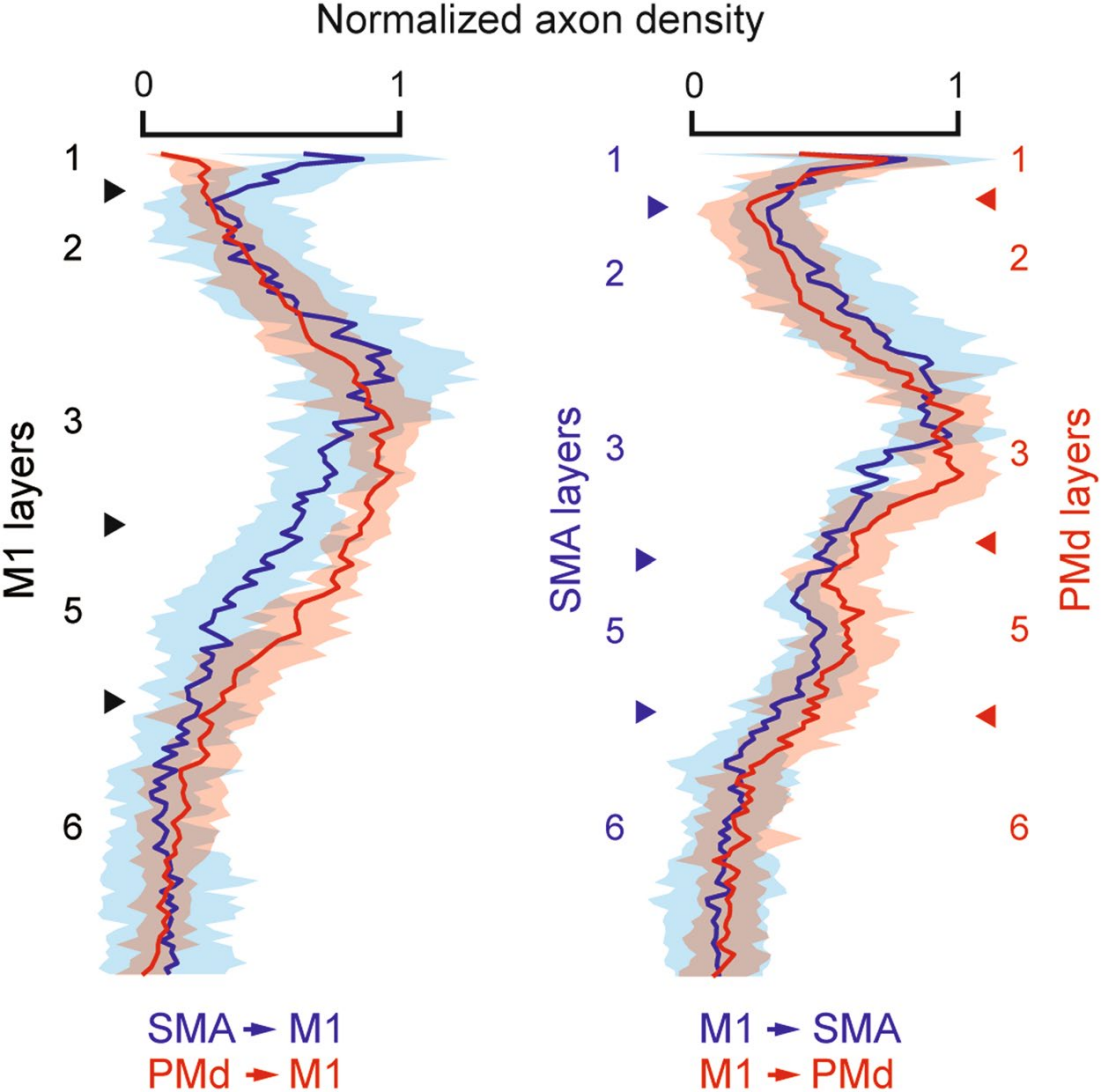
Anterograde and retrograde labeling in PMd after BDA injections into M1 (monkey A). See Fig. 6 for the result of ICMS mapping and the injection sites. All conventions and abbreviations are as in Figs. 1 and 4.

Finally, the laminar distributions of projections from M1 to SMA and PMd were evaluated following the tracer injections into M1 (monkeys A and G; Table 1). A total of 15 and 17 sites were analyzed in SMA (8 sites in monkey A and 7 sites in monkey G) and PMd (10 sites in monkey A and 7 sites in monkey G), respectively. The injection site of BDA in monkey A was placed mainly in the surface of the precentral gyrus where the proximal forelimb was represented (Fig. 6A), as confirmed by ICMS mapping as well as in previous studies^31,32^. Terminal labeling in SMA occurred prominently in layer 2/3, especially in its deeper part, and to a lesser but substantial extent in layer 5 (Fig. 6B–D). Layer 1 also contained certain labeling. A similar and more marked anterograde labeling in the superficial and deep layers was evident in PMd of the same sample (Fig. 7). Dense accumulations of labeled terminals were seen in the deeper part of layer 2/3 and layer 5 with some extension into layer 6. Qualitatively the same results were obtained in the case of WGA-HRP injections into the forelimb region of M1 (monkey G; data not shown).

Figure 3 summarizes the laminar distribution patterns of the intercortical connections investigated in the present study. While the projections from SMA and PMd both terminated predominantly in the superficial layers of M1, there were two noteworthy differences. First, the SMA projection ended in the upper part of layer 2/3 relative to the PMd projection. Indeed, when the density distribution of axon terminals in layer 2/3 was compared along the depth axis, the SMA projection was located significantly deeper than the PMd projection (t_88_ = 5.41, p = 5.33 × 10^−7^, two-tailed Welch’s t-test). Second, layer 1 seemed to be another principal target for the SMA input, but not for the PMd input. The percentage of layer 1 axon terminals for the SMA input was significantly larger than that for the PMd input (t_40_ = 5.86, p = 7.42 × 10^−7^, two-tailed Welch’s t-test). When the percentages of axon terminal distributions in the other layers were compared in the SMA vs. PMd inputs, those for the PMd input were significantly larger than those for the SMA input in the lower half of layer 2/3 (t_40_ = 3.52, p = 1.11 × 10^−3^, two-tailed Welch’s t-test) and the upper half of layer 5 (t_40_ = −2.51, p = 0.0164, two-tailed Welch’s t-test). There were no significant differences between the two inputs in the rest of the cortical layers (the upper half of layer 2/3, t_40_ = −0.865, p = 0.392; the lower half of layer 5, t_40_ = −0.475, p = 0.637; layer 6, t_40_ = −1.53, p = 0.134; two-tailed Welch’s t-test). On the other hand, the projections from M1 to SMA and PMd displayed a bilaminar pattern that was characterized by layers 2/3 and 5 terminations. The distributions of labeled terminals were statistically different from the ordinal Gaussian distribution (SMA, d = 0.532, p = 4.65 × 10^−37^, n = 146; PMd, d = 0.545, p = 9.06 × 10^−38^, n = 142, Kolmogorov–Smirnov test).

Retrograde labeling was also plotted in each section analyzed for anterograde labeling. In all of the injection cases, labeled neurons were observed in both the superficial and the deep layers. Specifically, in the SMA-injection cases (monkeys B and M), 63.4% (127/201) and 58.3% (230/395) of the labeled neurons were distributed in the superficial layers of M1. In the PMd-injection cases (monkeys B and H), 51.1% (332/650) and 60.7% (253/416) of the labeled neurons were found in the superficial layers of M1. In the M1-injection cases (monkeys A and G), 54.5% (103/189) and 53.9% (189/351) of the labeled neurons were located in the superficial layers of SMA, and 61.7% (186/302) and 53.8% (314/584) of labeled neurons were seen in the superficial layers of PMd. According to the criteria of Dum and Strick^27^, these laminar patterns are classified as “Equal” (33–67% of the labeled neurons in the superficial layers). Overall, these results are consistent with the data in the previous studies^21–27^. To rule out the possibility that the results obtained in this study were biased by the regions selected for quantification, all labeled neurons in SMA, PMd, and M1 were counted in every sixth section as a complementary analysis (see Table 2). The distribution of labeled neurons in each of the cases mentioned above was classified as “Equal”, indicating that the patterns of neuronal labeling in the selected regions were unbiased.

## Discussion

Employing dual anterograde tract-tracing, we have revealed the patterns of laminar connectivity among SMA, PM (especially PMd), and M1 in the present study. We have found that both SMA and PMd project chiefly to the superficial layers of M1, whereas M1 projects to SMA and PMd in a bilaminar manner. While gross laminar patterns of projections across the motor-related areas are available in previous studies^21,28–30^, we have shown that there are a couple of major differences in the laminar patterns of the projections among SMA, PMd, and M1. The peak density of SMA-derived terminals in M1 layer 2/3 shifts toward the upper part compared with that of PMd-derived terminals. Moreover, M1 layer 1 receives a strong input from SMA, but not from PMd.

**Figure 8:**
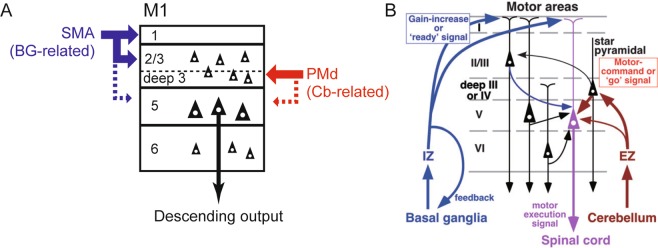
(**A**) Schematic diagram of projections from SMA and PMd to M1. The layer-specific inputs from SMA and PMd to M1 are shown in blue and red, respectively. Note that the input from SMA, functionally linked with the basal ganglia (BG), terminates mainly in layers 1 and 2/3 (upper part) and, to a lesser extent, in layer 5 (broken blue line) of M1, whereas the input from PMd, functionally relevant to the cerebellum (Cb), terminates predominantly in layer 2/3 (deeper part) and, additionally, in layer 5 (broken red line). (**B**) Schematic diagram of motor thalamocortical projections in rodents adopted from Kuramoto *et al*.^20^ In the ventral anterior and ventral lateral nuclei of the thalamus, the inhibitory afferent-dominant zone (IZ) and the excitatory afferent-dominant zone (EZ) relay BG-related and Cb-related signals to distinct layers of M1, respectively. Note the similarities of the layer-specific arrangements between SMA-derived and IZ-derived terminals, and between PMd-derived and EZ-derived terminals.

Of particular interest is that the difference in input terminations from SMA and PMd to M1 is in register with that in BG- and Cb-related thalamic inputs to M1 (Fig. 8). Although the thalamus relays nigral as well as pallidal inputs to frontal cortical areas, we here discuss the pallido-thalamo-cortical pathway since the forelimb region of M1, the target site in the current study, does not receive nigral input^18^. The pallidal input reaches M1 mainly via the oral division of the ventrolateral thalamic nucleus, while the cerebellar input is conveyed to M1 predominantly through the oral division of the ventroposterolateral thalamic nucleus, respectively^6,14,15,18^. Previous electrophysiological and anatomical studies indicate that no substantial convergence of the two inputs takes place in the thalamus^12,13,35,36^. Furthermore, M1 is most likely to receive BG- and Cb-related thalamic inputs in a layer-specific manner. It has been shown that layer 1 of M1 receives strong pallido-thalamo-cortical inputs across the species^11,16,17,19,20^. This laminar pattern of the projection well matches that of the projection from SMA to M1 as demonstrated in the present work. On the other hand, cerebello-thalamo-cortical input to M1 ends in both the superficial and the deep layers, with massive termination in layer 3^8–10,16,20^. Such a laminar organization of the cerebello-thalamo-cortical projection to M1 corresponds to that of the projection from PMd to M1. Although technical limitations in the current study do not allow us to identify neurons in M1 of which layer(s) actually receive input from SMA or PMd, it is possible to consider that BG and SMA or Cb and PMd send output to different populations of M1 neurons in the superficial layers, respectively, to generate signals for preparation and execution of a motor command, as also previously discussed by Kuramoto *et al*.^20^ (Fig. 8B).

The laminar origin and termination of corticocortical connections have been used as an indicator of the hierarchic relationships among cortical areas^27,37–43^. Felleman and Van Essen^37^ proposed a well-known scheme that classifies corticocortical connections into “ascending”, “descending”, and “lateral” according to the patterns of origin and termination in the cortical layers. In their scheme, a connection from one area to another is classified as “ascending” if it originates from the supragranular layers in a unilarminar manner or from the supragranular and infragranular layers in a bilaminar manner, and terminates in the input layer 4. A connection classified as “descending” arises from the infragranular layer in a unilaminar manner or from the supragranular and infragranular layers in a bilaminar manner, and terminates to avoid layer 4. A “lateral” connection originates in a bilaminar manner, but terminates in a columnar pattern across the layers. This classification is based primarily on the visual system, where the granular layer 4 is well developed. It may be problematic if one tries applying the scheme to the motor system as the motor-related areas are so-called agranular areas. Dum and Strick^27^ have reported that cortical motor neurons giving rise to their interconnections are distributed equally in the superficial and deep layers, which was confirmed with our dataset. The comparable laminar distribution of such interconnected neurons suggests that no bias exists in the direction of information flow among SMA, PMd, and M1, as previously concluded by Dum and Strick^27^. However, data obtained in the retrograde labeling studies alone are not necessarily sufficient to determine the hierarchic organization, because the bilaminar pattern of origin can be classified as any of “ascending”, “descending”, and “lateral”, as described above (see also refs. ^37,44^). In fact, the laminar distribution of SMA- and PMd-derived terminals in M1 exhibited a distinct pattern from that of M1-derived terminals in SMA and PMd. While both SMA and PMd send projection fibers mainly to the superficial layers of M1, M1 projects to the superficial and deep layers of SMA and PMd. Although we did find weaker labeling in the upper layer 5 of M1 in both the SMA- and the PMd-injection cases, there was no clear peak of terminal distribution in the deeper layers (see Fig. 3). Given that preferential termination in layer 3 is assigned as “ascending” in the agranular motor system^30,37^, a strong input to the superficial layer 3, but not to the deeper layers, identified in the SMA and PMd projections to M1 can be classified as “ascending”. On the other hand, the bilaminar projections from M1 to SMA and PMd are likely to be “lateral”. These patterns of cortical interconnections indicate that M1 is indeed posited at the higher level of hierarchy in the motor system. It remains debatable, however, if layer 3 of agranular areas is equivalent to layer 4 of granular areas, or if the “lateral” designation is applicable to connectivity within the motor system^44^. Furthermore, the classic view on M1 as being agranular has recently been challenged by some groups^45–47^. By examining sections of macaque M1 stained with Nissl and SMI-32, García-Cabezas and Barbas^46^ claimed that layer 4 can be identified as a thin and pale band between the bottom of large pyramidal neurons in layer 3 and the upper limit of giant pyramidal neurons in layer 5. The pale, sparsely populated band situated just above the upper limit of giant pyramidal neurons may probably correspond to the upper part of layer 5 (layer 5a) described in the studies of Matelli *et al*.^48^ and Geyer *et al*.^49^, the laminar parcellation in which we adopted in the present study. The ratios of neuronal labeling in the superficial vs. deep layers reported here would be changed if the laminar parcellation proposed by García-Cabezas and Barbas^46^ is adopted. However, since layer 4 (which is classified as layer 5 in the present study) does not belong to either the superficial or the deep layers, our conclusion that the projections from SMA to M1 and from PMd to M1 terminate primarily in the superficial layers remains valid. It should also be noted that even though SMA and PMd appear to send the “ascending” input to M1, these areas have significant corticospinal projections independent of the M1-derived projection^50^. Hence, it may be plausible to consider that the corticospinal pathway derived from M1 is not the “final common pathway” from the motor-related areas toward the spinal cord, but SMA and PMd play distinct roles with their direct corticospinal projections for the control of voluntary movement.

Taken together, the present results indicate that functionally-relevant signals (i.e., SMA/BG-related inputs and PM/Cb-related inputs) initially enter the same layers and then are integrated via local circuits within M1 to generate a motor command.

## Methods

The experimental protocols were approved by the Animal Welfare and Animal Care Committee of the Primate Research Institute, Kyoto University. All experiments were conducted in accordance with the Guide for Care and Use of Laboratory Primates (Ver. 3, 2010) issued by the institute. Five adult Japanese monkeys (*Macaca fuscata*, 4–8 kg, 5–9 years old) of either sex were used for this study. The monkeys were housed in individual cages in a 12-h light/dark cycle. They were fed a diet of commercial monkey chow supplemented with fruit. Water was available ad libitum. Details of the procedures for surgery, electrophysiological mapping, tracer injections, and histology were as described elsewhere^32,51,52^.

### ICMS mapping

All the tracer injections in this study were guided by electrophysiological mapping using ICMS technique. For this purpose, a head holder and a recording chamber were implanted under aseptic conditions prior to mapping. Each monkey was sedated with ketamine hydrochloride (10 mg/kg b. wt., i.m.) and then anesthetized with sodium pentobarbital (25 mg/kg b. wt., i.v.). The head was fixed in a stereotaxic apparatus. Anchor screws were implanted into the skull and a recording chamber was placed after a craniotomy guided by the stereotaxic coordinates of the target area. Two plastic pipes were then mounted in parallel over the frontal and occipital lobes as a head holder. The monkeys were given an analgesic (Lepetan; Otsuka, Tokyo, Japan; i.m.) and an antibiotic (Viccillin; Meiji Seika, Tokyo, Japan; 40 mg/kg/day for 5 days, i.m.) after the surgery.

After full recovery from the surgery, ICMS mapping was conducted to identify the target area for each experiment. In each mapping session, the monkey was lightly sedated with ketamine hydrochloride (5 mg/kg b. wt., i.m.) and positioned in a monkey chair. For extracellular unit monitoring, a glass-insulated Elgiloy-alloy microelectrode (impedance 0.5–1. 2 MΩ at 1,000 Hz) was inserted with a hydraulic micromanipulator attached to the stereotaxic frame. Trains of 11 or 44 pulses (up to 40 μA with 200-μs duration) were delivered at 333 Hz using the same microelectrode at every 500–1000 μm depth for ICMS mapping. Body movements and muscle twitches elicited by ICMS were examined by visual inspection and direct muscle palpation to determine the current threshold.

### Anterograde tract-tracing

After the ICMS mapping, cortical sites were chosen for injections of WGA-HRP or BDA for anterograde tract-tracing. We primarily selected the forelimb regions of SMA, PMd, and M1. A 4% solution of WGA-HRP (Toyobo, Osaka, Japan; diluted in 0.1 M Tris–HCl buffer, pH 7.0) or a 20% solution of BDA (Molecular Probes, Eugene, OR, USA; 1:1 mixture of 3,000 MW and 10,000 MW in 0.1 M phosphate buffer, pH 7.3) was injected into the target area by pressure through a 1-μL or 10-μL Hamilton microsyringe, respectively. The tracer injections were made into 2–3 sites for each targeted area (see Table 1). Typically, for each injection site, the tracer was deposited at two different depths (3.5 and 5 mm from the surface in the SMA- and M1-injection cases; 2.5 and 4 mm from the surface in the PMd-injection cases).

After survival periods of 3–4 days for WGA-HRP or >28 days for BDA, the monkeys were anesthetized deeply with sodium pentobarbital (50 mg/kg b.wt., i.v.) for perfusion-fixation. The monkeys were transcardially perfused with phosphate-buffered saline (0.1 M, pH 7.4), followed by fixatives. The fixative for the monkey with WGA-HRP injections was 8% formalin in phosphate buffer (PB; 0.1 M, pH 7.4) and the one for the monkeys with BDA injections was a mixture of 10% formalin and 15% saturated picric acid in PB. The brains were removed from the skull, postfixed in the same fresh fixative overnight, and saturated with 30% sucrose for 2 weeks at 4 °C. Coronal sections were cut serially at 60 μm thickness on a freezing microtome. In the case of WGA-HRP injections, a series of every sixth section was reacted with tetramethylbenzidine (TMB) and incubated for 30 minutes in a 3% solution of ammonium molybdate to stabilize the TMB reaction product. In the case of BDA injections, a series of every sixth section was initially treated with 0.3% hydrogen peroxide in 0.1 M phosphate-buffered saline (PBS; pH 7.4) for 30 minutes at room temperature to inhibit endogenous peroxidase. Subsequently, the sections were incubated with avidin-biotin-peroxidase complex (ABC Elite; 1:200 dilution; Vector Laboratories, Burlingame, CA, USA) in PBS with 0.1% Triton X-100 for 2 hours at room temperature. The sections were then reacted in 0.05 M Tris-HCl buffer (pH 7.6) containing 0.04% 3,3′-diaminobenzidine tetrahydrochloride, 0.04% nickel chloride, and 0.006% hydrogen peroxide. For both cases, another series of sections were Nissl-stained with 1% Neutral red or 1% Cresyl violet to visualize laminar and areal borders.

Laminar organizations of anterograde labeling were analyzed in M1 after SMA and PMd injections, and in SMA and PMd after M1 injections. Each varicosity or punctate filling labeled with BDA or WGA-HRP, respectively, was plotted by using brightfield microscopy with the Neurolucida computer-aided microscope system (MicroBright Field, Williston, VT, USA). Retrogradely labeled neurons were also plotted with the aid of the same system. Sites of tracing were targeted around regions where axonal labeling was most dense. In each cortical area, a total of 15 to 22 sites were analyzed, with at least 1.5 mm width for each site. Basically, we did not choose multiple sites from single sections, which ensured that any sites we traced were at least 360 μm apart from each other. To calculate a normalized density plot across the sections and the subjects for each area, the data obtained from anterograde tracing were processed according to the following procedure. First, plotted terminals in each section were binned along the depth axis (20 μm/bin) and normalized from 0 (no labeled terminal) to 1 (maximal number of terminals). Then, to obtain a “standard” laminar thickness of each layer, the averaged thickness was calculated across the analyzed sections. The binned data were then adjusted along the depth axis to the standard thickness of each layer. Finally, the adjusted data were averaged across the sections and the subjects and normalized from 0 (no labeled terminal) to 1 (maximal number of terminals) to reveal the laminar patterns of projections.

### Cortical parcellation

Laminar boundaries in each area were determined according to the studies of Matelli *et al*.^48^ and Geyer *et al*.^49^. Briefly, M1 is characterized by its poor lamination with a high concentration of giant pyramidal neurons in the deep part of layer 5 (layer 5b). The upper part of layer 5 (layer 5a) is poor in well-stained neurons. Granular layer 4 is not clearly visible in Nissl-stained sections. The laminar pattern of PMd is similar to that of M1: poor lamination, the absence of granular layer 4, and the presence of giant pyramidal neurons in layer 5. However, the decrements in the density and the size of the giant pyramidal neurons are apparent in this area. The SMA is also poorly laminated, but this area has higher cellular densities in the lower part of layer 3 and layer 5a compared with M1. Giant pyramidal neurons in layer 5 are absent or arranged in only a single row.

Parcellation of SMA, PMd, and M1 was based mainly on the differences in the laminar patterns as described above and in previous studies^53–57^. The results of the ICMS mapping were also utilized to determine the areal borders. The M1 and the other areas can be distinguished by the marked differences in thresholds to elicit body part movements^32,53–57^. In most cases of our experiments, microstimulation with a train of 11 pulses was effective only in the caudal part of the precentral gyrus and the rostra1 bank of the central sulcus, which correspond to the cytoarchitectonically defined M1 region.

## Data Availability

The datasets generated and/or analyzed during the current study are available from the corresponding author on reasonable request.
